# The Response of Hemostatic Marker Levels to Activated Factor VII in a Neonate following Cardiopulmonary Bypass

**DOI:** 10.1155/2009/420152

**Published:** 2009-08-31

**Authors:** Michael J. Eisses, Michael Richards, Denise Joffe, Jeremy M. Geiduschek, Wayne L. Chandler

**Affiliations:** ^1^Department of Anesthesiology and Pain Medicine, Seattle Children's Hospital, University of Washington School of Medicine, 4800 Sand Point Way NE, Seattle, WA 98105, USA; ^2^Department of Laboratory Medicine, University of Washington School of Medicine, 1959 NE Pacific Street, Seattle, WA 98195, USA

## Abstract

The primary function of recombinant activated factor VII (rFVIIa) is to increase thrombin formation which leads to increased fibrin and less “bleeding.” As a result, most of literature utilizes “bleeding” as the outcome measure with respect to rFVIIa. However, we report the actual effect of rFVIIa on changes in hemostatic markers such as prothrombin activation peptide F1.2, thrombin antithrombin complex (TAT), D-dimer, tissue plasminogen activator (tPA), and plasminogen activator inhibitor (PAI) in a neonate after cardiopulmonary bypass. A single dose of rFVIIa caused a 5.5-fold increase in F1.2, 3.5-fold increase in TAT, and a small increase in d-dimer compared to only a 1.5-fold increase, no increase, and a decrease, respectively, in two neonates undergoing the same procedure having not received rFVIIa. The patterns of change for tPA and PAI were similar.

## 1. Introduction

Recombinant activated factor VII (rFVIIa) is approved for use to treat bleeding due to hemophilia, especially patients with known inhibitors to typical factor replacement therapy [1]. Despite its approved use, rFVIIa has gained attention for its “off-label” uses which include treatment of bleeding due to trauma or surgery, reversal of warfarin therapy, and treatment of coagulopathies due to liver failure [2]. With regards to trauma and surgery, the medical literature reporting its “off-label use” includes mostly case reports or retrospective reviews with the predominant endpoint or outcome measure, consisting of a change in bleeding amounts or blood product administration. With regards to coagulopathies and warfarin reversal, outcome measures often include prothrombin time, which is heavily influenced by factor VII levels. Other parameters such as partial thromboplastin time and thrombin time have been included. However, the main purpose of rFVIIa administration in surgery or trauma is to increase thrombin formation which then catalyzes the conversion of fibrinogen to fibrin to make clot. In searching PubMed, we found no reports of rFVIIa on hemostatic markers such as F1.2 or TAT. In this case report, we present the direct changes in several hemostatic markers (F1.2, TAT, D-dimer, active tPA, and active PAI) due to the administration of rFVIIa in a neonate during cardiac surgery.

## 2. Case Presentation

A 2-week-old 2.5 kilogram neonate carrying the diagnosis of d-transposition of the great arteries underwent an arterial switch procedure using cardiopulmonary bypass (CPB). The patient had been consented to participate in an investigational review board approved research study involving the collection of hemostatic markers evaluating the changes in coagulation and fibrinolysis during CPB. The methods of that study have been published elsewhere [3]; however, none of the patients in this case report were included in previous publications. In brief, blood samples were collected at various time points during and after surgery, and levels of the following markers were obtained at each time point: prothrombin activation peptide F1.2, thrombin antithrombin complex (TAT), D-dimer (DD), active tissue plasminogen activator (active tPA), and active plasminogen activator inhibitor type 1 (active PAI-1). After the patient had separated from CPB and had received protamine to reverse the effects of heparin, a blood sample was obtained as part of the research protocol. One hour after that sample was obtained, this patient received a dose of rFVIIa (90 mcg/kg or 225 mcg) for continued bleeding despite platelet and plasma transfusions. Surgery was concluded successfully, and as part of the protocol, another blood sample was obtained one hour after the end of surgery in the pediatric intensive care unit (PICU). The same marker levels were evaluated. Using the blood sample after protamine administration as a baseline, the sample obtained in the PICU then demonstrated the effects of the rFVIIa administration. We also include in this report results from two other patients in the same study with the same age, diagnosis, and procedure who did not receive rFVIIa as a comparison. All patients did receive a one time dose of aprotinin in the CPB pump prime, but none received any infusion or subsequent doses. The timing of the blood samples was the same in all three patients.

The patient receiving rFVIIa showed a substantial increase in both markers of thrombin generation: F1.2 and TAT. While the two patients not receiving rFVIIa showed increases of around 1.5 to 1.8 times baseline, the patient receiving rFVIIa showed an almost 5.5-fold increase in F1.2. For TAT, the two non-rFVIIa patients showed little to no change while the patient receiving rFVIIa showed a 3.5-fold change. The pattern of D-dimer changes was different in that the patient receiving rFVIIa showed a small increase rather than a decrease as shown in the other two patients. Active tPA showed similar readings in the 3 patients, and active PAI-1 showed a smaller increase in the rFVIIa-treated patient compared to the others. See Table 1 for the complete results.

## 3. Discussion

Normally, when the endothelium of a blood vessel is disrupted, the thrombophilic subendothelial layer becomes exposed. Platelets begin to plug the hole by binding with vonWillebrand factor leading to activation and then further aggregation with other platelets through fibrinogen ligands. Upon this framework, stable clot is achieved through thrombin formation which then converts the fibrinogen ligands to fibrin. Factor Xa which is the catalyst for thrombin formation can be made through two different pathways, one involving a complex with activated factor VII and tissue factor (TF) and the other involving a complex with activated factors IX (FIXa) and VIII (FVIIIa). The amount of thrombin made from endogenous factor VII complexed with TF is small in comparison to the amount through complexed FIXa/FVIIIa. However, when recombinant FVIIa is given, the formation of thrombin shifts to predominantly the FVIIa/TF complex pathway, and therefore, factors IX and VIII become less important. This explains why recombinant FVIIa works in hemophiliac patients with factor VIII or IX inhibitors. 

The important regulatory substance to clot formation is thrombin. Not only does it serve as the catalyst to convert fibrinogen to fibrin but it also regulates the stimulation of factors XI, IX, and VIII to drive its own formation. Thrombin also further stimulates platelets and impacts activation of tissue plasminogen activator which is the initiation substance of the fibrinolytic system that will control the size of the clot being made. To measure the degree of this stimulation, many studies in literature utilize markers such as prothrombin activation peptide (F1.2) and thrombin antithrombin complex (TAT). In the case of F1.2, this peptide is released from the conversion of prothrombin to thrombin. Once thrombin is made, it is inhibited by antithrombin III making the TAT complex. Therefore both of these markers become a measure of thrombin activation, the former, directly, as thrombin is made, and the other indirectly through inhibition of thrombin. 

Because of the mechanism of recombinant FVIIa, we felt it prudent to report its effect on these more focused hemostatic markers. In this case, the large increase in markers of thrombin generation (F1.2 and TAT) was striking. Generally, it has been shown that after protamine administration and into the first moments after completion of cardiac surgery a procoagulant state may already predominate as represented by increased F1.2 and TAT [4]. That type of response was dramatically increased as a result of rFVIIa. In the case of D-dimer (a breakdown product of fibrin), we previously reported [3] that its typical response after cardiac surgery in infants is to drop in the postoperative period. However, the one infant that received rFVIIa showed an increase in D-dimer postoperatively. One can speculate that this may be due to the increased fibrin formation associated with increased thrombin generation. There was little difference in the typical drop in active tPA postsurgery in the patient receiving rFVIIa. Finally, while active PAI has been shown to increase dramatically postsurgery, the patient receiving rFVIIa showed a less dramatic increase. Reasons for this are unclear. 

While most reports on rFVIIa in literature center around outcome measures such as blood loss, in this case we report its effect on common outcome markers of coagulation and fibrinolysis. Ultimately, an understanding of how rFVIIa affects rates of thrombin and fibrin formation and how it affects other systems such as fibrinolysis or inflammation will need further study and perhaps modeling techniques.

## Figures and Tables

**Table 1:** Changes in Marker levels from before and after rFVIIa. nM: nanomolar. pM: picomolar. F1.2: prothrombin activation fragment. tat: thrombin antithrombin complex. dd: d-dimer. apai: active plasminogen activator inhibitor type 1. atpa: active tissue plasmingoen activator. TP8: Time point 8, after protamine or baseline for this report. TP9: Time point 9, 1 hour after surgery or after rFVIIa, if applicable.

Patient receiving rFVIIa?	F1.2 (nM)		TAT (pM)		DD (pM)		apai (pM)		atpa (pM)	
	TP8 baseline	TP9	TP8 baseline	TP9	TP8 baseline	TP9	TP8 baseline	TP9	TP8 baseline	TP9

No	3.8	6.9	505	514	4419	3222	218.7	1524.5	73.4	1.3
No	2	3	356	342	7371	5401	4.5	1013.3	120.8	2
Yes	5	27	536	1927	2364	3081	80.4	453.1	105.8	4.2
